# A case of papillary thyroid carcinoma with renal and pulmonary metastases

**DOI:** 10.1093/jscr/rjac366

**Published:** 2022-08-13

**Authors:** Mariko Aoyama, Tomohiro Inui, Naoki Miyamoto, Shinichi Sakamoto, Seiya Inoue, Satoshi Fujiwara, Masakazu Goto, Hiroaki Toba, Hiromitsu Takizawa

**Affiliations:** Department of Thoracic, Endocrine Surgery and Oncology, Institute of Health, Biosciences, The University of Tokushima, Tokushima, Japan; Department of Thoracic, Endocrine Surgery and Oncology, Institute of Health, Biosciences, The University of Tokushima, Tokushima, Japan; Department of Thoracic, Endocrine Surgery and Oncology, Institute of Health, Biosciences, The University of Tokushima, Tokushima, Japan; Department of Thoracic, Endocrine Surgery and Oncology, Institute of Health, Biosciences, The University of Tokushima, Tokushima, Japan; Department of Thoracic, Endocrine Surgery and Oncology, Institute of Health, Biosciences, The University of Tokushima, Tokushima, Japan; Department of Thoracic, Endocrine Surgery and Oncology, Institute of Health, Biosciences, The University of Tokushima, Tokushima, Japan; Department of Thoracic, Endocrine Surgery and Oncology, Institute of Health, Biosciences, The University of Tokushima, Tokushima, Japan; Department of Thoracic, Endocrine Surgery and Oncology, Institute of Health, Biosciences, The University of Tokushima, Tokushima, Japan; Department of Thoracic, Endocrine Surgery and Oncology, Institute of Health, Biosciences, The University of Tokushima, Tokushima, Japan

**Keywords:** papillary thyroid carcinoma, renal metastasis, pulmonary metastasis, radioactive iodine therapy, lenvatinib, immune checkpoint inhibitor

## Abstract

Distant metastases derived from papillary carcinoma are generally detected in the lungs and bones. However, renal metastasis is rare. We herein report a case of papillary thyroid carcinoma with renal and pulmonary metastases that had been initially diagnosed as primary renal carcinoma with pulmonary metastases. The lesions showed response to immune checkpoint inhibitors and tyrosine kinase inhibitor but not to radioactive iodine therapy.

## INTRODUCTION

Distant metastases derived from papillary carcinoma (PTC) occur in ~4% of patients and are generally detected in the lungs and bones [1]. However, renal metastasis is rare.

We herein report a case of PTC with extensive renal and pulmonary metastases treated with multimodal therapy. The lesions were also treated with immune checkpoint inhibitors because they had been initially diagnosed as primary renal carcinoma with pulmonary metastases.

## CASE REPORT

**Figure 1:**
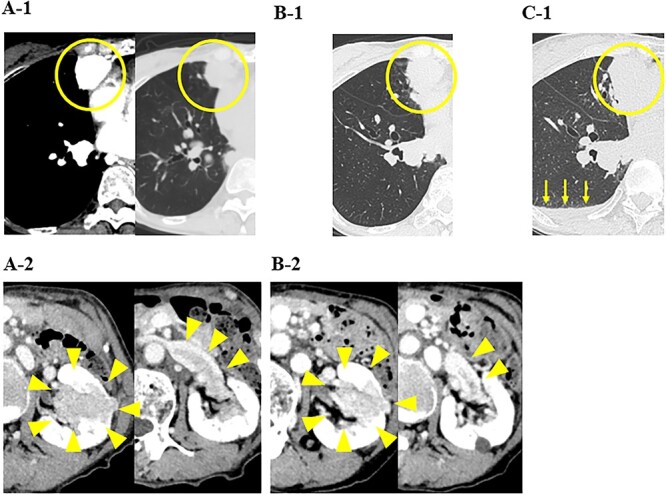
CT images of the chest and abdomen; (**A**) at the start of the administration of nivolumab and ipilimumab, CT showed multiple masses in both lung fields and a mass in the left kidney with a tumor plug in the left renal vein, and (**B**) 5 months after the administration of nivolumab and ipilimumab, CT showed increases in the sizes of the pulmonary tumors and a decrease in the size of the renal tumor, and (**C**) 6 months after the administration of nivolumab and ipilimumab, CT showed increases in the sizes of the pulmonary tumors and pleural effusion (yellow arrows).

**Figure 2:**
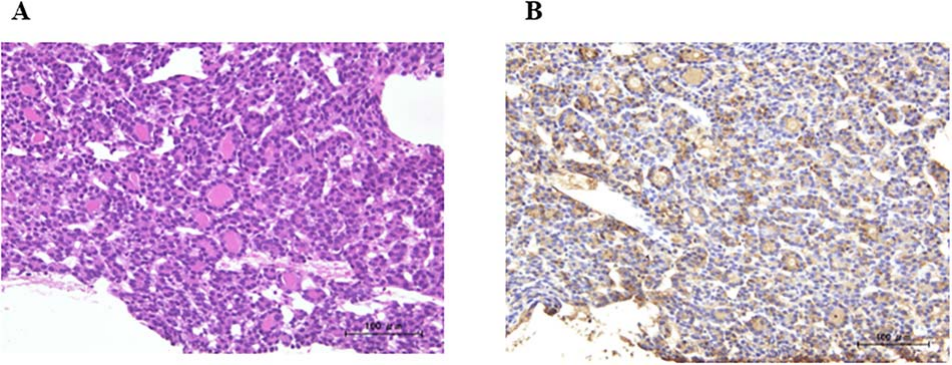
A pathological examination of the pulmonary mass; (**A**) tumor cells formed a papillary structure, and the lumen was filled with colloids (H. E. stain), and (**B**) immunohistochemistry results were positive for Tg.

A79-year-old female underwent right thyroid lobe resection and cervical lymph node dissection for PTC at a previous hospital. Five years after the initial surgery, follow-up computed tomography (CT) revealed multiple masses in both lung fields, with the largest tumors in the middle lobe of the right lung measuring 2.6 cm. These tumors previously showed a high signal intensity in the early phase of contrast-enhanced CT (Fig. 1A-1). A mass of ~4 cm was detected in the left kidney with a tumor plug in the left renal vein (Fig. 1A-2). From the CT findings and clinical course, primary renal cell carcinoma (RCC) with multiple pulmonary metastases were strongly suspected, and the administration of nivolumab and ipilimumab was initiated. Five months later, CT showed increases in the sizes of the pulmonary tumors (Fig. 1B-1) and a decrease in the size of the renal tumor (Fig. 1B-2).

In our hospital, CT-guided core needle biopsy was performed on the pulmonary mass of the right middle lobe. At the time of biopsy, which was performed ~1 month after the last CT scan, increases in the sizes of the pulmonary tumors (tumor growth rate of pulmonary tumors; 29%) (Fig. 1C-1) and pleural effusion (Fig. 1B-1) were noted on CT. A pathological examination revealed that the tumor formed a papillary structure and the lumen was filled with colloids. Since immunohistochemistry results were positive for Tg (Fig. 2), the mass was considered to be pulmonary metastasis of PTC. The patient was diagnosed with pulmonary metastases of PTC, and the administration of lenvatinib was initiated. Since nivolumab and ipilimumab were administered until just before the initiation of lenvatinib, the starting dose of lenvatinib was 8 mg/day. Fatigue (grade 2) and skin disorders (grade 2) developed 3 weeks after the initiation of lenvatinib, and thus, the dose was reduced to 4 mg/day. Three weeks after the dose reduction, hypertension (grade 2) was observed. The patient was administered olmesartan medoxomil, which returned blood pressure to normal.

**Figure 3:**
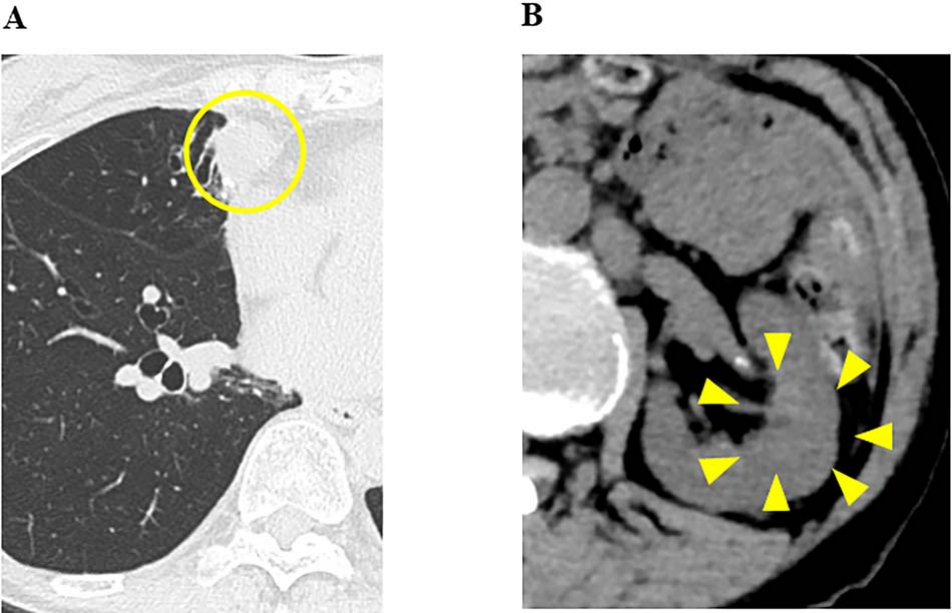
CT images of the chest and abdomen 10 months after the start of the administration of lenvatinib, and (**A**) pulmonary metastases decreased in size; (**B**) the size of the renal tumor decreased and the tumor plug disappeared.

**Figure 4:**
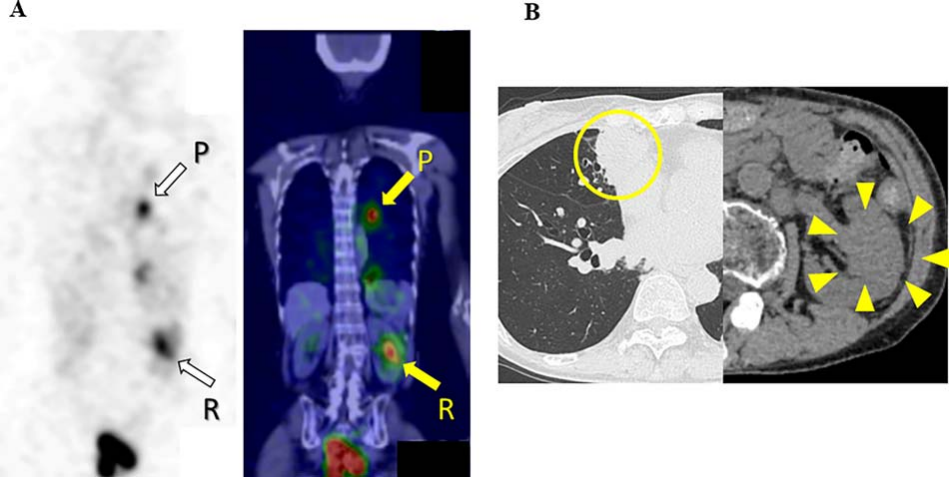
131I scintigraphy and CT images of the chest and abdomen after the second surgery; (**A**) in 131I scintigraphy, some lung metastases showed radioiodine uptake, whereas others did not. Radioiodine uptake was also observed in the left renal tumor, but not in the right kidney; left: RAI scintigraphy, right: SPECT–CT, P: pulmonary metastases, R: renal metastasis; (**B**) pulmonary and renal metastases both increased in size.

Ten months after the start of lenvatinib, CT showed a decrease in the sizes of pulmonary metastases (Fig. 3A) and renal metastasis (Fig. 3B) (tumor reduction rate: 45%). Remnant thyroid resection was performed and followed by RAI therapy with 100 mCi. In 131I scintigraphy, some lung metastases showed radioiodine uptake, whereas others did not. Radioiodine uptake was also observed in the left renal tumor, but not in the right kidney (Fig. 4A); therefore, the left renal tumor was finally confirmed as renal metastasis of PTC. Since the sizes of pulmonary and renal metastases both increased after the RAI therapy (tumor growth rate: 71%) (Fig. 4B), the administration of lenvatinib was resumed from a dose of 4 mg/day. After resuming lenvatinib, she has been in SD status for 6 months.

## DISCUSSION

The clinical detection of differentiated thyroid carcinoma (DTC) renal metastasis is rare, with a prevalence of 0.47% [2]. The renal metastasis of DTC is generally associated with multifocal metastasis and appears several years after surgery for primary thyroid carcinoma. CT and magnetic resonance imaging do not have specific findings for metastatic renal tumors originating from thyroid carcinoma; therefore, difficulties are associated with reaching a differential diagnosis between primary renal cancer and renal metastasis of DTC based on imaging findings alone [3].

Ten percent of patients with thyroid carcinoma are estimated to develop advanced disease that may become resistant to RAI therapy [4]. TKI were recently developed as a new therapeutic strategy for the treatment of RAI-refractory metastatic DTC. Based on the findings of the SELECT trial, lenvatinib has been approved for RAI-refractory metastatic DTC. [5]. In a phase 2 trial, patients with RCC showed a good response to lenvatinib [6].

In the present case, the patient was diagnosed as RCC with multiple pulmonary metastases, and the administration of nivolumab and ipilimumab was initiated. In our hospital, since the size of pulmonary metastasis rapidly increased, it was challenging to perform complete thyroidectomy followed by whole-body RAI scanning. Because lenvatinib was expected to be effective for both RCC and pulmonary metastasis of PTC, the administration of lenvatinib was started. And lenvatinib, which inhibits angiogenesis, was expected to be effective against metastatic lesions, which were very hypervascular on contrast-enhanced CT. The administration of lenvatinib provided long-term disease control and it was possible to perform remnant thyroid resection as well as whole-body RAI scanning. Based on which the renal tumor was diagnosed as metastasis of papillary thyroid carcinoma.

The first treatment for the recurrence and distant metastasis of DTC is generally RAI therapy. Riker reported that lenvatinib was used as initial treatment for a patient with advanced liver metastasis of thyroid papillary cancer liver metastasis before thyroidectomy and RAI [7]. Depending on the condition of distant metastatic organs, TKI treatment before RAI may be effect to obtain good disease control.

In the present case, the size of renal metastasis was reduced by nivolumab and ipilimumab. In KEYNOTE-028 study, pembrolizumab presented high tolerability in patient diagnosed with DTC that had progressed with standard therapy [8]. Immune checkpoint inhibitors have a potential to become one of the effective treatments for DTC. However, the size of pulmonary metastases increased. Further studies on a larger number of cases with long-term observations are needed to clarify the reasons for histological type-dependent differences in therapeutic effects.
